# Identification of a Novel CCM1 Frameshift Mutation in a Chinese Han Family With Multiple Cerebral Cavernous Malformations

**DOI:** 10.3389/fnins.2020.525986

**Published:** 2020-09-23

**Authors:** Fan Zhang, Yiteng Xue, Feng Zhang, Xiaoming Wei, Zhisong Zhou, Zhaoru Ma, Xiaosong Wang, Hong Shen, Yujun Li, Xiaoying Cui, Li Liu

**Affiliations:** ^1^Department of Anesthesiology, The First Affiliated Hospital of Harbin Medical University, Harbin, China; ^2^Department of Neurosurgery, The First Affiliated Hospital of Harbin Medical University, Harbin, China; ^3^Department of Microbiology and Wu Lien-Teh Institute, Harbin Medical University, Harbin, China; ^4^Queensland Brain Institute, The University of Queensland, St Lucia, QLD, Australia

**Keywords:** FCCMs, CCM1, frameshift deletion, truncated protein, hemorrhage

## Abstract

Cerebral cavernous malformations (CCMs) are vascular lesions that predominantly occur in the brain. CCMs can be sporadic or hereditary in an autosomal dominant manner. The genes harboring variants of familial CCMs (FCCMs) include CCM1/KRIT1, CCM2/MGC4607, and CCM3/PDCD10. In this study, we identified a novel CCM1/KRIT1 mutation in a Chinese family with FCCMs. This family consists of 20 members, and 6 of them had been diagnosed with CCMs. The proband patient is a 17-year-old female who has suffered from CCM-related intracranial hemorrhage four times. Magnetic resonance imaging (MRI) revealed four lesions in the different brain regions and one lesion has progressively enlarged. The pathological histology confirmed CCMs. Whole exome sequencing revealed a novel deletion mutation (c.1635delA) within exon 15 of CCM1/KRIT1 gene in the proband patient, her mother, and her uncle who had CCMs. This frameshift mutation led to a premature termination codon (PTC) at nucleotides 1652–1654. We also detected that the CCM1 mRNA levels in the blood lymphocytes of the family members with CCMs were reduced by 46.4% compared to that in healthy controls. Collectively, our results suggested that the CCM1 mutation could potentially be a causative factor for FCCMs in the Chinese family and the reduction of CCM1 mRNA expression in the blood lymphocytes of the patients might be a potential biomarker for the diagnosis and prognosis of CCMs. Our findings expanded the spectrum of CCM mutations and helped to guide genetic counseling and early genetic diagnosis for at-risk family members.

## Introduction

Cerebral cavernous malformations (CCMs) are abnormal clusters of small blood vessels that are primarily present in the brain. As the second most prevalent vascular malformations in the central nervous system, CCMs account for 10–15% of all vascular malefactions cases (Batra et al., 2009; Flemming et al., 2017). The majority of CCMs are sporadic, covering over 90% of all cases while familial CCMs (FCCMs) only account for 6–7%, which is considered as a rare disease (Moriarity et al., 1999a; Batra et al., 2009; Spiegler et al., 2018; Zafar et al., 2019). However, less than 20% of patients with sporadic CCMs have multiple lesions (Moriarity et al., 1999b; Dammann et al., 2017; Flemming and Lanzino, 2020), whereas over 50% of FCCMs cause multiple CCM lesions, which will increase the risk of developing epilepsy and intracranial hemorrhage (Russo et al., 2017; Zafar et al., 2019).

CCMs affect about 0.5% of the population, but almost 40% of patients are asymptomatic (Moore et al., 2014). With the advent of magnetic resonance imaging (MRI) and computerized tomography scan (CT), CCMs are always discovered incidentally during MRI scans (Porter et al., 1997; Morris et al., 2009; Zafar et al., 2019). Most patients developed CCMs at their 30s to 40s, but a quarter of CCMs take place in childhood and even during infancy (Al-Holou et al., 2012; Brinjikji et al., 2017) and thus dramatically impact on the quality of the patients’ life. The clinical manifestations of CCMs include epilepsy, headache, intracranial hemorrhage and focal neurological dysfunction (Denier et al., 2004; Al-Shahi Salman et al., 2012; Flemming and Lanzino, 2020). The clinical pathology has shown that within the CCM lesions, these blood vessels are often enlarged and lack tight endothelial cell junctions and vesicular smooth muscles. The irregular vessels are prone to leakage and cause cerebral hemorrhage (Frischer et al., 2008; Fischer et al., 2013; Zhou et al., 2016).

FCCMs are inherited in an autosomal dominant manner with incomplete penetrance. The specific pathogenic genes of FCCMs mainly include CCM1/KRIT1 (Krev1 interacting trapped gene), CCM2/MGC4607, and CCM3/PDCD10 (programmed cell death protein 10) (Dupre et al., 2003; Bergametti et al., 2005; Denier et al., 2006; Chang et al., 2019; Cianfruglia et al., 2019; Zafar et al., 2019). About 87–98% of patents with FCCMs have been found to carry mutations in one of these CCM genes (Denier et al., 2006; Spiegler et al., 2014). More than half (53–65%) of the inherited CCMs cases are related to CCM1/KRIT1 mutations, and the remaining cases are associated with the genetic variants in CCM2 (15–19%) and CCM3 (11–20%), respectively (Wang et al., 2017). The three CCM proteins form a protein complex that interacts with other proteins to regulate vasculogenesis and angiogenesis (Batra et al., 2009; Fisher et al., 2015a; Zhou et al., 2015, 2016). The functional roles of CCM proteins have been demonstrated using several mouse (Tang et al., 2017, 2019) and zebrafish models (Jin et al., 2007; Hogan et al., 2008; Kleaveland et al., 2009). For example, in CCM1 knockout mice, the vessel dilation in the brain was observed as early as embryonic day 8.5. The thin-walled and dilated vessels are reminiscent of human CCMs defects (Whitehead et al., 2004).

Both clinical and preclinical studies suggested that CCM mutants might be causal; hence, identification of pathogenic variants not only helps the early diagnosis of FCCMs but also lays the groundwork for understanding the molecular basis of CCMs. In this study, we identified a novel mutation of CCM1 that is associated with clinical and pathological phenotype of FCCMs in a Chinese Han family. Specifically, this mutation c.1635delA (p.Thr545fsTer6) was a deletion frameshift mutation in the CCM1/KRIT1 gene, which resulted in a premature translation termination. We examined the expression of CCM1 mRNA in the blood lymphocytes of the family members who carried CCMs. We considered that this CCM1 mutation could be a pathogenic factor for CCMs in the Chinese family.

## Subjects and Methods

### Subjects

A three-generation Chinese Han family with 20 members and three healthy individuals were included in this study. The study was approved by the ethics review committee of the First Affiliated Hospital of Harbin Medical University. All family members and healthy persons involved in this study had signed the informed consent forms.

### Clinical Characteristics

**FIGURE 1:**
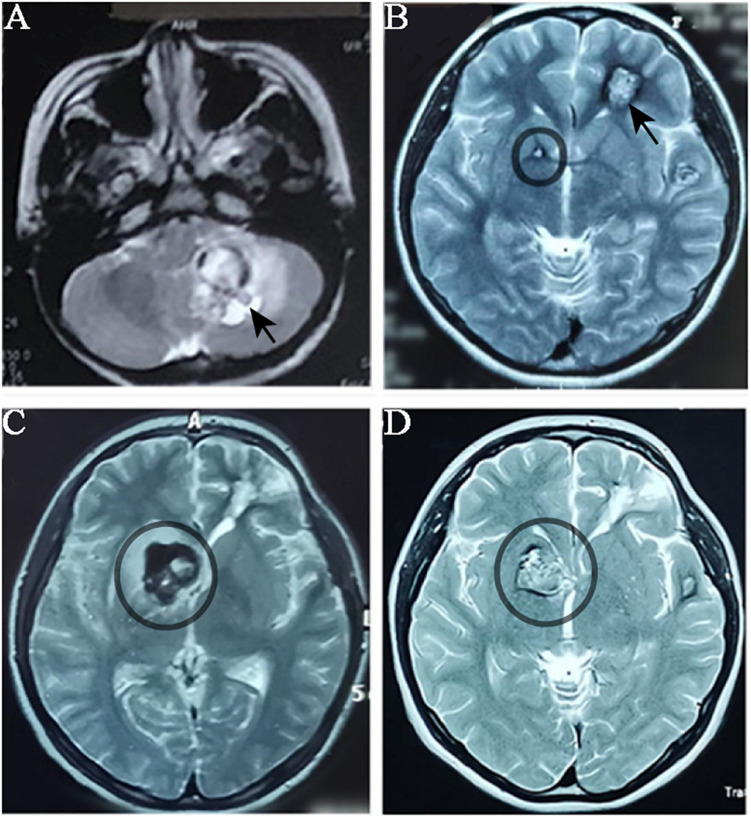
MRI imagology of the proband’s head during the progression of CCMs. The proband had CCMs-associated haemorrhage for four times over five years. MRI images were taken when the proband patient had haemorrhage. **(A)** MRI (T1-weighted) detected a lesion located in the left cerebellar hemisphere (black arrow) when the proband had her first haemorrhage. **(B)** When the proband had her second haemorrhage, MRI (T2 weighted) showed a lesion in the left frontal lobe (which was surgically removed) and a small lesion in right basal ganglia (black circle). **(C)** and **(D)** The lesion in right basal ganglia was obviously enlarged in two years.

**FIGURE 2:**
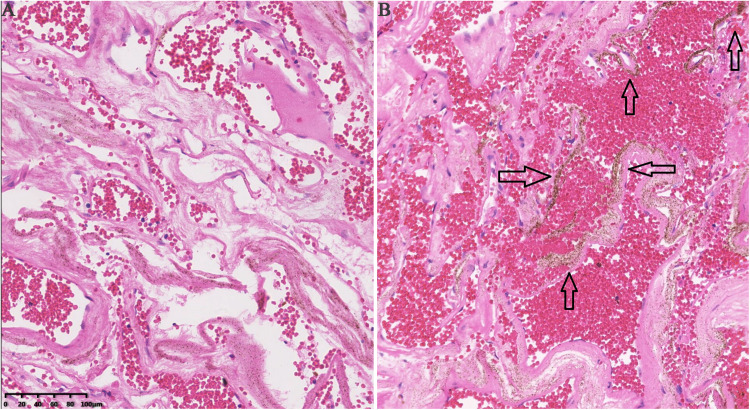
Pathological histology of CCMs specimen from the proband patient. **(A)** H&E staining demonstrated the back to back arrangement of the dilated vessels and the thin-walled capillaries that lacked of tunica media. **(B)** The hemosiderin pigments (blank arrows) were detected in surrounding tissues.

The proband was a 17-year-old female who had suffered CCM-related intracranial hemorrhages four times (Table 1). Firstly, she was hospitalized due to dizziness and vomiting symptoms after a slight head trauma. Head MRI showed multiple CCMs in the left cerebellar hemisphere, right temporal lobe, bilateral frontal lobe, and brain stem. The fourth ventricle was squeezed by the lesion in the left cerebellar hemisphere, and this hemorrhagic lesion was surgically excised (Figure 1A). Pathological diagnosis was CCM. Two years later, the proband experienced headache, dizziness, and vomiting after a head trauma again. MRI revealed abnormal signals in the left frontal lobe and right basal ganglia (Figure 1B). A left frontal CCM-related hemorrhage was diagnosed and the left frontal lesion was surgically removed. Pathological diagnosis was CCMs (Figure 2). In the next 3 years, the patient again suffered from CCM-related intracerebral hemorrhage twice. The lesion occurred at the same region of the right caudate nucleus head (Figures 1C,D). The proband and her family claimed no obvious inducement prior to the symptom onset. The patient only had some non-specific symptoms such as somnolence and mild headache. Considering high mortality and disability rate of operation, the patient was given a conservative medical treatment. During the follow-up, the head computed tomography (CT) showed that the hematoma was well absorbed (data not shown).

**TABLE 1:** The proband’s detailed conditions for the four times.

Order of onset	Age of onset (years)	Style of onset	Signs and symptoms	Location of onset	Inducement	Treatment
1st time	11.7	Hemorrhage	Dizziness and vomiting	Left cerebellar hemisphere	Slight head trauma	Operation
2nd time	14.3	Hemorrhage	Headache, dizziness and vomiting	Left frontal lobe	Head trauma	Operation
3rd time	16.0	Hemorrhage	Somnolence and slight headache	Right caudate nucleus head	None	Conservation
4th time	17	Hemorrhage	Somnolence	Right caudate nucleus head	None	Conservation

### Tissue Preparation and Histological Staining

CCM tissues were removed from the proband’s brain and fixed in formalin overnight at 4°C. The tissue was then embedded in paraffin wax and cut into 10-μm sections. Hematoxylin and eosin (H&E) staining was conducted using Harris-modified hematoxylin (Fisher Scientific) and alcoholic eosin (Fisher Scientific). The images were captured using Olympus FV-10 microscope.

### Whole Exome Sequencing

The blood samples were collected from three registered members (II5, II7 and III4) according to the standard procedures. Genomic DNA was extracted using PureLink Genomic DNA Mini Kit (Invitrogen, United States). DNA quality was examined using the 2100 Bioanalyzer System. Genomic DNA was sheared into 150–200 bp using the Bioruptor Pico (Bioruptor, United States). Illumina libraries were prepared with the Fast Library Prep Kit according to the manufacturer’s protocol (iGeneTech Co., Ltd.) and subject to the Illumina HiSeq 2500 sequencing system (iGeneTech Co., Ltd). Prior to bioinformatic analysis, the raw reads were filtered and removed to avoid problems in the following analysis according to the following criteria: (1) containing of adapter reads, (2) low-quality reads, which were defined as a base quality ≤5, and (3) containing unknown bases >10%. The “clean reads” were used for bioinformatic analysis. Sequencing data were analyzed using MiSeq Reporter (MSR) software. Burrows–Wheeler Aligner (BWA) was used to align the reads against the hg19 *Homo sapiens* reference genome to create BAM files. The Genome Analysis Toolkit (GATK) was used for variant analysis in the target regions. The basic quality statistics was shown in Supplementary Tables S1, S2. The identified variants/mutations were compared with the known mutations in ClinVar, human gene mutation database (HGMD), or ExAC database (GnomAD database).

### Real-Time Quantitative Polymerase Chain Reaction (qPCR)

The qPCR assay was performed as described previously (Li et al., 2013). Total RNA was isolated from the blood samples (2 ml) collected from the proband (III4) and two FCCM family members (II5 and II7) and three healthy controls using Trizol (Invitrogen). Five hundred nanograms of total RNA per sample was used for cDNA synthesis using PrimeScript^TM^ RT reagent Kit with gDNA Eraser (Takara Bio, Japan) according to the manufacturer’s protocol. The PCR reaction volumes were 20 μl containing 5 μl of cDNA, 5 μl of primer mix (0.5 μM as final concentration), and 10 μl of TB Green^TM^ *Premix Ex Taq*^TM^ II (Takara Bio, Japan). The PCR was 45 cycles of 95°C for 15 s, 60°C for 20 s, and 72°C for 20 s. The results were analyzed using the comparative threshold method. The relative levels of CCM1 mRNA were normalized to the mean value of two housekeeping gene glyceraldehyde 3-phosphate dehydrogenase (GAPDH) and hypoxanthine-guanine phosphoribosyltransferase (HPRT). The primers were purchased from GENEWIZ (Suzhou, China). Sequences for the primers were listed in Table 2.

**TABLE 2:** Primer sequences.

Gene	Sequence (5′ to 3′)
CCM1 Forward	ATGCGAGTCTGTAGTGAATCCA
CCM1 Reverse	TGTGCATGACGTTCATCTAACC
GAPDH Forward	CTCACCGGATGCACCAATGTT
GAPDH Reverse	CGCGTTGCTCACAATGTTCAT
HPRT Forward	TGACCAGTCAACAGGGGACA
HPRT Reverse	GCGACCTTGACCATCTTTGG

## Results

### Family Pedigree

**FIGURE 3:**
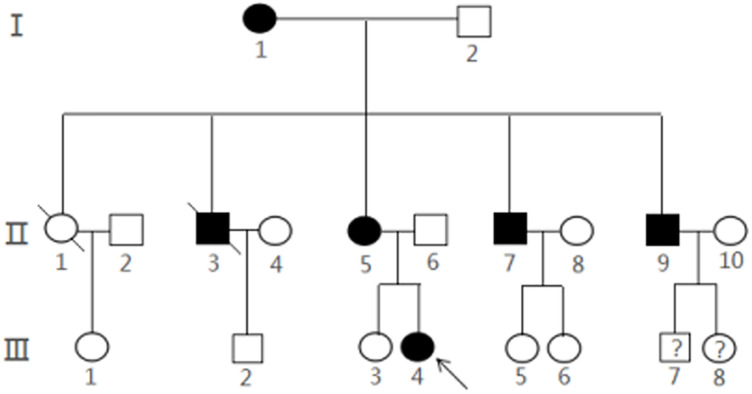
Pedigree of a Chinese family with CCMs. The arrow represented the proband III4 and filled symbols represented family members with CCMs. Squares indicated males, and circles indicated female. An oblique line through the symbol demonstrated a deceased individual.Interrogation point represents that it is unknown whether the subject is a CCMs carrier.

With the help of the proband and her parents, the pedigree was established (Figure 3). There were 20 members in this three-generation family (12 females and eight males). Six of them (three females and three males) were diagnosed with multiple CCMs using MRI, while the proband (III4) and one of her uncles (II7) had ongoing CCM-related intracranial hemorrhage. Another uncle (II3) died of accident. Other three CCM carriers (I1, II5, and II9) were asymptomatic. Two members of the family, III-7 and III-8, were not examined systematically because of their young ages.

### Mutation Analysis

**FIGURE 4:**
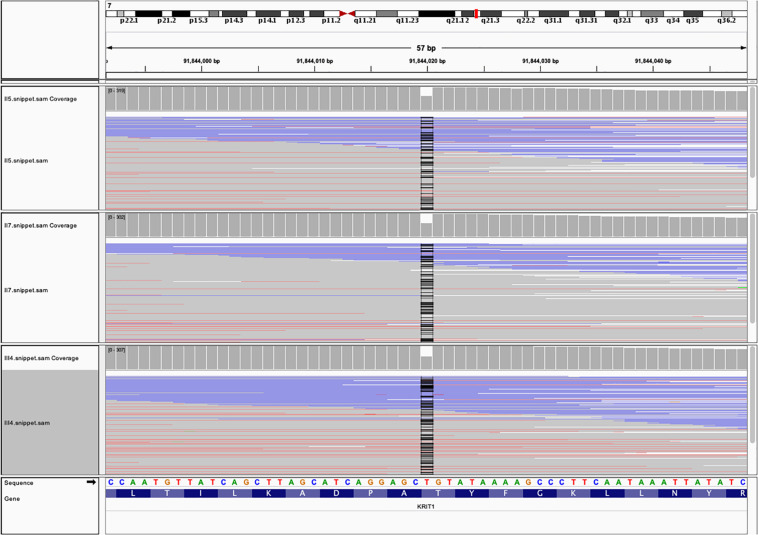
The whole exome sequencing (WES) data from the three family members with FCCMs. A chromosome ideogram with the genetic location of CCM1 (red line) was shown in the upper part [(hg19) chr7:92,198,968 – 92,246,100]. Read alignment of the hybrid capture WES data from the proband III4, her mother II5 and her uncle II7 showed a part of exon 15of CCM1 gene. The coverage plot indicated that no read was found at position of chr7:91,844,020 (black line).

Next-generation sequencing identified a deletion-frameshift mutation c.1635delA (p.Thr545fsTer6) in the exon 15 of CCM1 gene (20 exons in total) in proband (III4), her mother (II5), and her uncle (II7) (Figure 4). This mutation changed the CCM1 gene reading frame, which led to a premature termination codon (PTC) at nucleotides 1652–1654. We then validated this mutation using Sanger sequencing (Supplementary Figure S1). This variant was not listed in the ClinVar, human gene mutation database (HGMD), or ExAC database (GnomAD database).

### Quantitative Analysis of CCM1 mRNA Expression

**FIGURE 5:**
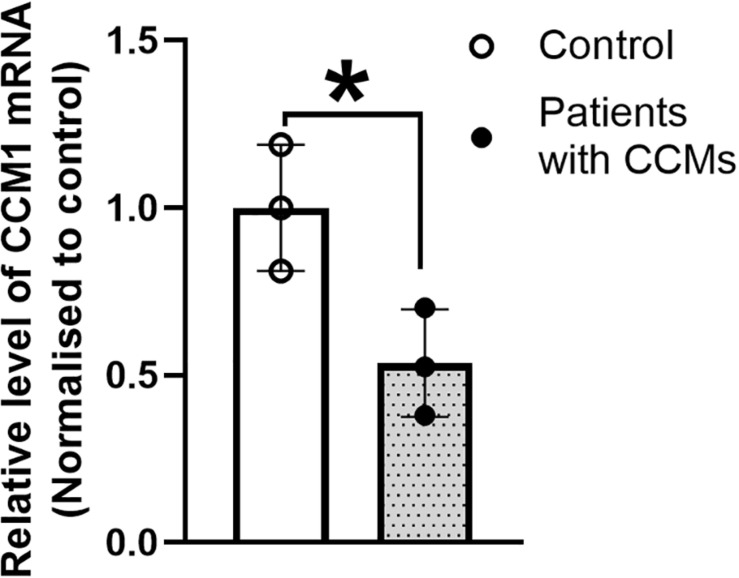
The expression of CCM1 mRNA in the three patients from a Chinese family with FCCMs. Real time PCR result showed a significant reduction of CCM1 mRNA in the blood lymphocytes of the three patients (The proband III4, her mother II5 and her uncle II7) compared with three healthy controls. Data = mean ± SEM, *p < 0.05.

Real time qPCR results showed that the CCM1 mRNA expression was decreased by 46.4% in the blood lymphocytes of the family members with CCMs compared with that in the normal individuals (Figure 5). This reduction was significant (unpaired *t* test with Welch’s correction, *t* = 3.240, *df* = 3.903, *p* < 0.05).

### Protein Structure of KRIT1

As CCM1 frameshift mutation (c.1635delA) resulted in a PTC, the mutated transcripts of CCM1 was predicted to undergo degradation rapidly, a process named non-sense-mediated mRNA decay (NMD). NMD is a translation-coupled process to remove the mRNAs containing PTC, which had been found in multiple disorders (Schwarze et al., 2001; Brogna and Wen, 2009). Alternatively, the mutated transcript might be stabilized by polyadenylation, which might produce a truncated protein lacking 191 (546–736) amino acids at the C-terminal of CCM1 protein. The reduction of CCM1 mRNA in the blood lymphocytes of the family members with CCMs suggested that mutated transcript of CCM1 might degrade, and thus, truncated protein might not exist or be present at a very low level. We predicted the structure of the truncated protein. The deletion region was positioned in the band 4.1/ezrin/radixin/moesin (FERM-3) domain of KRIT1 protein (Supplementary Figure S2).

## Discussion

In this study, we identified a deletion frameshift mutation c.1635delA (p.Thr545fsTer6) in CCM1 gene in a Chinese Han family. Six members of this family carried multiple CCMs. CCM1 mutation resulted in a significant reduction in CCM1 mRNAs in the blood lymphocytes of the family members with CCMs. As CCM1 plays a critical role in endothelial cell junctions of blood vessels, we speculated that this CCM1 mutation could be a causative factor for CCMs in the Chinese family.

Gene variants of FCCMs are found in three genes, CCM1, CCM2, and CCM3 (Morrison and Akers, 2003 [updated 2016 Aug 4]; Spiegler et al., 2018; Zafar et al., 2019). To date, more than 200 germline genetic variants in CCM1–3 have been unraveled as causative factors for the etiology of CCMs (Scimone et al., 2017). In Chinese population, about 21 CCM gene variants were identified (summarized in Supplementary Table S3). Similar to the previous research, most genetic variants were found in CCM1 genes. The current study identified a novel frameshift mutation in CCM1 (c.1635delA) in a Chinese family with CCMs. In this three-generation family with 20 family members, about 30% of them (six members) have developed CCMs with multiple lesions that are the common features of FCCMs (Zafar et al., 2019; Flemming and Lanzino, 2020). The positive family history in which CCMs are present in the proband, her maternal grandmother, her mother, and her uncles indicated an autosomal dominant inheritance. The proband (III4), her mother (II5), and her uncle (II7) carried this frameshift CCM1 mutation. Collectively, we tentatively concluded that the novel CCM1 multination could be the pathogenic gene for CCMs in this Chinese family.

Similar to the known CCM mutations, the CCM1 mutation (c.1635delA) identified in this study resulted in a PTC. Our qPCR results showed a significant reduction of CCM1 mRNA in the blood lymphocytes of the family members with CCM1. This suggested that the CCM1 truncated transcripts might undergo NMD. Cave-Riat et al. screened 121 unrelated CCM probands who had one or multiple lesions and found that 43% of them carried CCM1 mutations. Importantly, all of these CCM1 mutations led to PTC. NMD-mediated degradation of mRNA containing PTC was considered as a potential mechanism of CCMs (Cave-Riant et al., 2002). To draw a solid conclusion, the presence of a KRIT1 truncated protein needs to be examined when specific antibodies against KRIT1 N-terminal is available.

Normal CCM1/KRIT1 contains 20 exons and KRIT1 protein is composed of a Nudix domain, three Asn-Pro-X-Tye/Phe (NPX/Y/F) motifs, an ankyrin domain, and a FERM domain at the C-terminal. There are three subdomains (F1–F3) in the FERM domain (residues 426–726) (Serebriiskii et al., 1997; Liu et al., 2013; Fisher et al., 2015b). Our structural modeling predicted that the CCM1 mutation-induced deletion localized within the FERM-3 domain (residues 545–726). We suspected that this deletion in the FERM-3 domain might impair CCM1 function since FERM domains are the key mediator for the interaction between CCM1 and the junctional proteins that are required for maintenance of tight endothelial junctions of blood vessels. For example, KRIT1 is localized at the endothelial cell junction via its FERM domain that can also bind to Ras-related protein Rap1, a small GTPase. KRIT1 acted as a specific effector of Rap1, which stabilized the cell–cell junction and regulated the endothelial permeability (Glading et al., 2007). In addition, Gigrass et al. have shown that the transmembrane protein heart of glass (HEG1) interacted with FERM domain to recruit KRIT1 protein to the cell junction of blood vessel (Gingras et al., 2012). Taken together, it is plausible that our novel CCM1 mutation could cause a large deletion within the FERM-3 domain, which lacks its ability binding to Rap1 or HEG1 and thus leads to an impairment in the endothelial cell junctions and an increase in the permeability of the blood vessels. This could be a potential linkage between the CCM1 mutation and CCM phenotype in this family. However, the presence of CCM1 truncated protein was warranted to be examined in future studies.

Furthermore, the FERM domain is also required to maintain the normal levels of KRIT1 protein, as deletion of the FERM domain reduced KRIT1 protein expression by 50% (Spiegler et al., 2014). We detected a significant reduction of CCM1 mRNA in the family member with CCMs, which might be attributed to the FERM domain deletion. In line with our finding, Mao et al. also reported that a mutation c1159 G > T within the FERM domain resulted in a decrease (35%) in CCM1 expression in a Chinese family (Mao et al., 2016). Nevertheless, a preclinical study has shown that deletion of KRIT1 in the endothelial cells induced hypersprouting and multiple CCMs in CCM1 knockout mouse brain (Mleynek et al., 2014). The decreased expression of KRIT1 increased the vascular permeability in the heterogeneous KRIT1 knockout mice (Corr et al., 2012). Therefore, we predicted that CCM1 mutation-induced downregulation of CCM1 mRNA might potentially result in multiple CCM formation and increase vascular permeability in this Chinese family.

One interesting finding in this study is that the course of the CCMs varied significantly between individuals that share the same CCM1 mutation in this family. The onset age of the proband is only 12 years old, who had suffered CCM-related hemorrhage four times, while her mother who carries the same CCM1 mutation and had multiple CCMs is asymptomatic. This phenomenon has been observed in many other studies (Lucas et al., 2001; Spiegler et al., 2014; Fauth et al., 2015). There is a second hit hypothesis that the patient with CCMs could carry not only a germline mutation but also a somatic mutation, which might contribute to the progression and severity of the disease. However, the somatic mutation was not assessed in the current study, which is warranted in future studies.

## Conclusion

In summary, we identified a novel deletion-frameshift mutation in CCM1 in a Chinese Han family with CCMs. Our finding enriches the mutation gene database of CCMs. Identifying mutations within CCM genes will pave the way for understanding the molecular basis of CCMs. Furthermore, with the development of advanced genetic editing tools, future research might target the pathogenic mutations to prevent the occurrence or progression of CCMs.

## Data Availability Statement

The original contributions presented in the study are publicly available. This data can be found here: https://www.ncbi.nlm.nih.gov/sra/PRJNA650147.

## Ethics Statement

The studies involving human participants were reviewed and approved by The First Affiliated Hospital of Harbin Medical University. Written informed consent to participate in this study was provided by the participants’ legal guardian/next of kin. Written informed consent was obtained from the individual(s), and minor(s)’ legal guardian/next of kin, for the publication of any potentially identifiable images or data included in this article.

## Author Contributions

FaZ, YX, and LL designed research, analyzed data, and drafted the manuscript. FeZ, XMW, ZZ, HS, and LL collected samples. ZM, XSW, and YL performed research. XC drafted and edited the manuscript. All authors contributed to the article and approved the submitted version.

## Conflict of Interest

The authors declare that the research was conducted in the absence of any commercial or financial relationships that could be construed as a potential conflict of interest.

## Supplementary Material

FIGURE S1 Sanger sequencing showed a deletion-frameshift mutation c.1635delA in the exon 15 of CCM1 in the patients of FCCMs family.

FIGURE S2 Structure of the truncated CCM1 protein. CCM1 mutant c.1635delA results in a truncated protein that is lack of partial Ferm_3 domain. The gray region is the missing segment.

TABLE S1 The quality control statistics of whole exome sequencing of the blood from the patients with FCCMs.

TABLE S2 The basic statistics of whole exome sequencing of the blood samples from family members with FCCMs.

TABLE S3 Known CCM mutations in Chinese population.
