# Supplementary material for: Identification of a Novel CCM1 Frameshift Mutation in a Chinese Han Family With Multiple Cerebral Cavernous Malformations
Source: Front Neurosci. 2020 Sep 23;14:525986. doi: 10.3389/fnins.2020.525986 (PMC7538688; doi:10.3389/fnins.2020.525986)
Supplement: FIGURE S1 — Sanger sequencing showed a deletion-frameshift mutation c.1635delA in the exon 15 of CCM1 in the patients of FCCMs family. [file Presentation_1.pdf]

Supplementary method and results

Supplementary method

Sanger sequencing

Genome DNA was isolated using PureLink Genomic DNA Mini Kit (Invitrogen, USA) from the blood samples of the patients. 200 ng genome DNA was used to amplify the CCM1 gene across exon 14 and exon 15. The primers were: forward primer 5'-GGCGCTAAAACATCCTGTCAAAA - 3' and reverse primer 5'-TTGGCTCCACATCTGATTGC -3'. PCR was conducted using Takara LA PCR TM kit （Takara） according to the manufacture's protocol and the products were subject to sequencing.

Protein structure analysis

3D structure of the truncated CCM1/KRIT1 protein was predicted by the SWISS-MODEL server and illustrated using Swiss-pdb viewer software (Version 4.10).

Supplementary Results

Supplementary Figure 1. Sanger sequencing showed a deletion-frameshift mutation c.1635delA in the exon 15 of CCM1 in the patients of FCCMs family

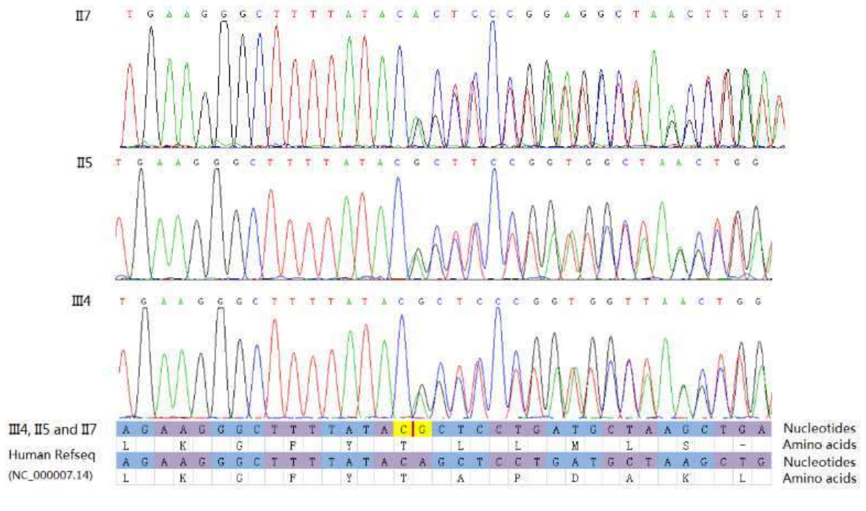

Supplementary Figure 1. Sanger sequencing showed a deletion-frameshift mutation c.1635delA in the exon 15 of CCM1 in the patients of FCCMs family. The mutation was detected in the proband III4, her mother II5 and her uncle II7 (red line).

Supplementary Figure 2. Structure of the truncated CCM1 protein

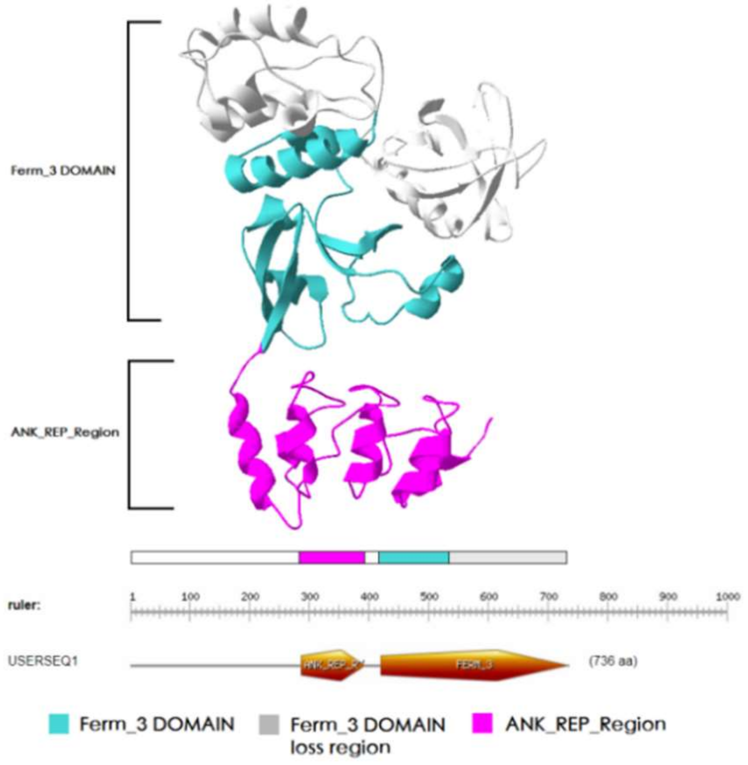

Supplementary Figure 2. Structure of the truncated CCM1 protein. CCM1 mutation c.1635delA resulted in a truncated protein that lacked of partial Ferm_3 domain. The grey region represented the missing segment.

Supplementary Table 1. The quality control statistics of whole exome sequencing of the blood samples from the patients with FCCMs

Name	III4	II5	II7
Raw reads (M)	80.69	110.95	107.5
Raw bases (Mb)	12102.71	16643.08	16124.69
Raw Q20-R2 (%)	97.15	97.36	98.05
Clean reads (M)	79.29	109.11	105.93
Clean bases (Mb)	11348.91	15660.0	14988.24
Clean Q20-R2 (%)	98.27	98.33	98.76

Supplementary Table 2. The basic statistics of whole exome sequencing of the blood samples from family members with FCCMs.

Name	III4	II5	II7
Total mapped reads(M)	78.93	106.12	105.58
Total reads mapping rate (%)	99.55	97.26	99.67
Target mapped reads (M)	52.1	63.23	87.17
Target reads capture rate (%)	66.01	59.58	82.56
Target mean depth	119.72	144.99	184.5
T 4X coverage rates (%)	99.61	99.84	99.81
T 10X coverage rates (%)	99.37	99.62	99.68

T 20X coverage rates (%)	98.75	99.14	99.42
T 30X coverage rates (%)	97.56	98.41	98.97

Supplementary Table 3. Known CCM mutations in Chinese population

	Author	Gene	Exon/intron	Nucleotide change	Mutation consequence	Predicted amino acid change
2002	Chen et al	CCM1/KRIT1	Exon 18	c.2092C>T	Nonsense	p.Gln698X
2003	Xu et al.	CCM1/KRIT1	Exon 13	c.1289C>G	Nonsense	p.Ser430X
2003	Mao et al.	CCM1/KRIT1	Exon 13	c.1292_1293delAT	Frameshift	p.Tyr431SerfsX4
2004	Xie et al.	CCM1/KRIT1	Exon 12	c.1160A>C	Missense	p.Gln387Pro
2005	Xie et al.	CCM1/KRIT1	Exon 8	c.704_705insT	Frameshift	p.Ser236IlefsTer11
2006	Ji et al.	CCM1/KRIT1	The acceptor splicing site in intron 12/exon 13	c.1255-4_1255-2delGTA	Exon 13 skipping or frameshift	p.Tyr419AlafsX24, or p.Leu516TrpfsX11
2010	Lan et al.	CCM1/KRIT1		c.1708A>T	Nonsense	P.Lys570Ter
2011	Zhao et al.	CCM1/KRIT1	Exon 12	c.1197_1200delCAAA	Frameshift	p.Gln401ThrfsX10
2013	Wang et al.	CCM1/KRIT1	Exon 13	c.1396delT	Frameshift	p.Cys466ValfsX29
2014	Zhu et al.	CCM1/KRIT1	Exon 14	c.1542delT	Frameshift	p.Leu516TrpfsX11
2016	Mao et al.	CCM1/KRIT1	Exon 14	c.1159G>T	nonsense	p. Glu387Ter
2016/7	Yang et al.	CCM1/KRIT1	Exon 16	c.1780delG	Frameshift	p.Ala594HisfsX67
2016	Yang et al.	CCM1/KRIT1	The acceptor splicing site in intron 13	c.1412-1G>A	Exon 14 skipping or frameshift	p.Ser471AsnfsX2 or p.Ser471ThrfsX24
2016	Huang et al.	CCM2/MGC4607	Exon 2	c.95 del C	Frameshift	p. Ala32fsTer4
2017	Yang et al.	CCM1/KRIT1	Exon 17	c.1864C>T	Nonsense	p.Gln622X
2017	Wang et al.	CCM1/KRIT1	Exon 18?	c.1896_1897insT	Frameshift	p.Pro633SerfsTer22
2017	Chang et al.	CCM1/KRIT1	Exon 18	c.1846delA	Frameshift	p.Glu617LysfsTer44
2018	Wang et al.	CCM1/KRIT1		c.1599_1601TGAdel	Deletion	p.Asp533del
		CCM2/MGC4607		c.773delA	Frameshift	p.K258fsX34
2019	Yu et al.	CCM3/PDCD10	Exon 4	c.212delG	Frameshift	p.S71Tfs*18
2019	Du et al.	CCM2/MGC4607	Exon 2	c.55C>T	Missense	p. R19X
2020	Our study	CCM1/KRIT1	Exon12	c.1635delA	Frameshift	p.Thr545fsTer6
